# Resting-State Networks Associated with Behavioral and Self-Reported Measures of Persecutory Ideation in Psychosis

**DOI:** 10.3390/brainsci11111490

**Published:** 2021-11-11

**Authors:** Lingyan Yu, Rebecca Kazinka, Danielle Pratt, Anita Kwashie, Angus W. MacDonald

**Affiliations:** Department of Psychology, University of Minnesota, Minneapolis, MN 55455, USA; yu000341@umn.edu (L.Y.); kazin003@umn.edu (R.K.); pratt308@umn.edu (D.P.); kwash001@umn.edu (A.K.)

**Keywords:** persecutory ideation, psychosis, resting-state networks, prefrontal

## Abstract

Persecutory ideations are self-referential delusions of being the target of malevolence despite a lack of evidence. Wisner et al. (2021) found that reduced connectivity between the left frontoparietal (lFP) network and parts of the orbitofrontal cortex (OFC) correlated with increased persecutory behaviors among psychotic patients performing in an economic social decision-making task that can measure the anticipation of a partner’s spiteful behavior. If this pattern could be observed in the resting state, it would suggest a functional-structural prior predisposing individuals to persecutory ideation. Forty-four patients in the early course of a psychotic disorder provided data for resting-state functional connectivity magnetic resonance imaging across nine brain networks that included the FP network and a similar OFC region. As predicted, we found a significant and negative correlation between the lFP–OFC at rest and the level of suspicious mistrust on the decision-making task using a within-group correlational design. Additionally, self-reported persecutory ideation correlated significantly with the connectivity between the right frontoparietal (rFP) network and the OFC. We extended the previous finding of reduced connectivity between the lFP network and the OFC in psychosis patients to the resting state, and observed a possible hemispheric difference, such that greater rFP–OFC connectivity predicted elevated self-reported persecutory ideation, suggesting potential differences between the lFP and rFP roles in persecutory social interactions.

## 1. Introduction

Persecutory ideation is a self-referential delusion where individuals believe that they are being targeted by malicious actors despite a lack of evidence. It is important for our understanding of both psychiatric and normative populations because it is the most common type of delusion found in psychotic disorders [1], and to large degrees in the general population [2]. As such, understanding the biological mechanisms of persecutory ideation may provide insights into delusions in general. In clinical settings, persecutory ideation often interferes with therapeutic alliance and treatment compliance, which makes understanding its mechanisms critical for optimizing clinical outcomes [3]. To understand the proclivity of interpreting stimuli as threatening in this manner, this study examined a behavioral marker of persecutory decision making while the brain was at rest.

Functional neural networks are traditionally studied during tasks or with explicit instructions. Recently, more researchers have used resting-state networks, or RSNs, to predict brain activation patterns during tasks. This approach follows findings that the voxels making up networks continuously covary during tasks, rest, sleep, and even under anesthesia [4]. Not only are the networks spatially consistent across subjects but the signal amplitude is also consistent with those observed under tasks. RSNs may therefore reflect a “functional-structural prior” where internal perturbations activate existing network configurations spontaneously in the absence of external stimuli [5]. Thus, functional neural networks show a clear correspondence between task and rest, and can be used as an identification measure for individuals [6].

Resting-state neuroimaging is particularly beneficial as resting-state data collection involves less manipulation and parameter setting, and it is easy to standardize across different studies. This makes study results more comparable and more conducive to meta-analysis. As a neural identifier, RSNs are also sensitive to neurological and psychiatric disorders [7,8,9,10]. Deviations from the general pattern may, therefore, provide insight and even markers for specific symptoms.

The functional connectivity literature in schizophrenia, for example, has reported aberrant interactions within and between the default mode network (DMN, anatomically the medial frontoparietal network) and the central executive network (anatomically the lateral frontoparietal network) [11,12]. DMN abnormalities, in particular, were found to be associated with the severity of delusion [13]. Decreased connectivity in the left striatum was found to correlate with increased severity of delusions as well [14]. Along with decreased activation of the right anterior insula in schizophrenia [15], Nekovarova et al. [16] proposed the triple network dysfunction theory, arguing that the dysfunctional switching between the DMN and the central executive network was modulated by the impaired salience network (SN, anatomically the anterior insula, dorsal anterior cingulate cortex, and striatum). However, none of this previous work focused on RSNs that characterize more persecuted patients.

For this study we built on work using the task-based network connectivity reported by Wisner and colleagues [17]. This study investigated the neural correlates of persecutory ideation using an economic social decision-making task. The SN was found to be sensitive to suspicious mistrust and the orbitofrontal cortex (OFC)/ventromedial prefrontal cortex (vmPFC) to the difference between suspicious and rational mistrust. Moreover, the authors found that higher persecutory ideation predicted reduced functional connectivity between the OFC/vmPFC and the left frontoparietal (lFP) networks. We hypothesized that similar patterns would be observed with the resting-state data: in a newly acquired independent sample, psychosis patients high in persecutory ideation would show reduced connectivity between OFC/vmPFC and the lFP network during rest. We then explored the association between persecutory ideation and the connectivity of other functional networks. 

We adopted a within-patient design in order to dismantle the specific neural signature of the symptom of persecutory ideation. While psychopathology research traditionally used a pseudo-experimental paradigm that treated DSM diagnoses as an experimental manipulation, this approach has been increasingly challenged in recent years. The Research Domain Criteria [18,19] and the Hierarchical Taxonomy of Psychopathology (HiTOP) model [20] both highlight the importance of transdiagnostic research by recognizing that symptoms shared across a number of artificial categories likely share common rather than distinct mechanisms. While traditional case-control studies were optimized to distinguish the average patient from the average control, they failed to account for heterogeneity within diagnoses. Systematic confounds such as education, medication status, history of hospitalization, global functioning, and often the history of experiencing bias contribute to the observed differences between patients and healthy controls as well. Within-patient studies, on the other hand, are optimized for sensitivity to a specific symptom, either within or across diagnoses. Heterogeneity, rather than being a source of noise, can provide the signal. While within-patient studies are not without risks—for example, any given symptom also contributes to a patient’s overall severity—the general background that everyone in the sample is experiencing some symptoms helps reduce systematic confounds that have historically biased case-control studies.

For the reasons outlined above, we performed the current analyses on a group of early psychiatric patients with a history of psychosis using a within-patient group design. We predicted that Independent Component Analysis (ICA) components comprising the lFP and the OFC/mPFC would show resting-state functional connectivity. Among those who showed this pattern, we predicted that persecutory ideation would negatively correlate with the resting-state functional connectivity between the lFP and portions of the OFC network.

## 2. Materials and Methods

### 2.1. Participants

Fifty-four patients with psychotic symptoms were recruited via a patient advocate from the First Episode Psychosis Program under the University of Minnesota Psychiatry Department. All participants were between 18 and 45 and spoke sufficient English to comprehend the study. They were phone screened and excluded from the study if they had any fMRI contraindications, changes in medication immediately prior to the study, current substance and/or alcohol intoxication on the day of the study, met the criteria for substance use disorder in the past 3 months, or had an estimated IQ below 70 (i.e., scoring below 6 on the Wechsler Test of Adult Reading; WTAR) [21]. Three participants were thus excluded for scanning contraindications, one for cannabis intoxication, and one for low estimated IQ. Five more participants would be excluded from analyses for missing more than 30% of good-quality resting-state data (see Resting-State Functional Neuroimaging Data section for details). The final sample for analysis was 44 participants.

Participants’ decision-making capacity was confirmed via the University of California, San Diego Brief Assessment of Capacity to Consent (UBACC) [22] before they consented to the study. On the day of the visit, the consent was reviewed, and participants were screened for MR safety a second time. Participants were screened for current drug and/or alcohol use via urine test and breathalyzer. Participants were then administered the Mini-International Neuropsychiatric Interview [23] and self-reported questionnaires for psychotic symptoms. The patients included fifteen individuals with a diagnosis of schizophrenia, twelve with schizoaffective disorder, seven with mood disorders with psychotic features, nine with mood disorders without psychotic features currently, and two who did not fulfill these diagnostic criteria.

Participants were briefly trained to ensure comprehension of a decision-making task, which they completed partially outside of the scanner and partially inside. They were compensated for their time and had the opportunity to be paid a smaller amount of money based on the decisions they made during the task.

### 2.2. Methods

#### 2.2.1. Behavioral Measure of Persecution: Minnesota Trust Game (MTG)

MTG is an economic social decision-making task where participants are instructed to choose between an assured payoff and a payoff determined by an unknown partner. The paradigm was developed to measure suspicious personality through individuals’ patterns of mistrust in choices where their partner had no monetary incentive to betray. Participants are presented with the choice between an assured payoff, where they and their partners both get USD 10, or they can let their partners choose between (1) a win-win where both get USD 20 or (2) a temptation. If the partner selects the temptation, the player will be left with an “adverse payoff” which ranges from losing USD 15 to winning USD 22 in USD 1 increments. In the Suspiciousness condition, the partner’s temptation is USD 15, therefore incentivizing the partner to select the mutually rewarding USD 20 payoff. When participants choose the assured payoff over allowing their partner to choose, they are displaying suspicion that their partner is spiteful [17,24]. In the Rational Mistrust condition, the partner’s temptation is USD 25, incentivizing the partner to leave the participant with the adverse payoff. The latter condition was not of interest to our study. For our analyses, two outcomes from the Suspiciousness condition were taken as the behavioral measures of persecutory ideation. First, we calculated the percentage of no trust choices, and then we calculated a threshold in which the player switched from trusting to not trusting based on the adverse payoff, using a Heaviside function.

#### 2.2.2. Self-Reported Measures of Persecution: Green et al. Paranoid Thought Scales (GPTS)

GPTS is a 32-item self-reported questionnaire from which two factors may be extracted. The first factor summarizes ideas of reference, and the second is about ideas of persecution. In this study, we used the persecution scale as the measure for self-reported persecutory ideation. The scales were selected for their dimensionality and high psychometric quality [25].

### 2.3. Resting-State Functional Neuroimaging Data

Participants were scanned in a Siemens 3T scanner with a 32-channel head coil at the University of Minnesota’s Center for Magnetic Resonance Imaging. A high-resolution T1-weighted scan was obtained for registration (repetition time (TR) = 2.5 s; echo time (TE) = 1.8 ms; flip = 8°; voxel = 0.8 × 0.8 × 0.8 mm). The resting-state data were collected in two whole-brain echo-planar imaging runs in the anterior-to-posterior and posterior-to-anterior directions to correct for field distortion (TR = 800 ms, TE = 37 ms, flip angle = 52°, voxel size = 2 × 2 × 2 mm, multiband factor = 8). A total of 400 volumes were collected in each run, totaling a little less than 11 min of resting-state data with two directions combined.

**Figure A1:**
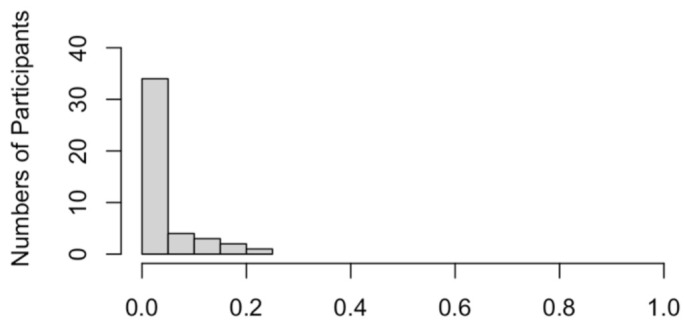
Motion in the sample of analysis (N = 44) after removing participants with less than 70% of resting-state data below the motion threshold (0.3 rms/0.5 FD). These data then underwent motion regression using the same threshold.

The preprocessing pipeline included MCFLIRT (FMRIB’s intra-modal motion correction tool), interleaved slice timing correction, spatial smoothing (5 mm), and high-pass temporal filtering (0.01 Hz). The resting-state functional data were registered to the T1 anatomical image using boundary-based registration. The data were then warped into the MNI152 standard space (degree of freedom = 12). Following Siegel et al. [26], we selected a more stringent motion threshold at 0.3 root mean square (rms) or 0.5 frame displacement (FD). Volumes with motion above this threshold were regressed out of the time series so that they exerted no influence on subsequent analyses. Participants included in the study had more than 70% of volumes below the motion threshold before regression (see Figure A1).

### 2.4. Data Analysis Plan

We conducted dual regression on the data using the predefined 60-component ICA map developed by Rueter and colleagues [27]. As both the ICA map and our resting-state data were already in the standard space, this step simply located the group-ICA components using the same coordinates in individual brains, from which we acquired the time series per ICA component per subject. Network analysis was conducted on the resulting time series for components that covaried with each other across time using FSLNets [28] on MATLAB. Functional connectivity between two components was calculated as the correlation of their time series, which then underwent Fisher’s transformation. The larger the resulting z-score was, the more positively correlated two components were and the stronger their functional connectivity was.

We first examined the normality of persecutory ideation measures to determine the appropriate tests, demeaned them, and then examined their correlations with each other and with participants’ characteristics. We treated persecution as a dimensional construct and used a within-group design to examine its neural correlates in patients. 

**Figure A2:**
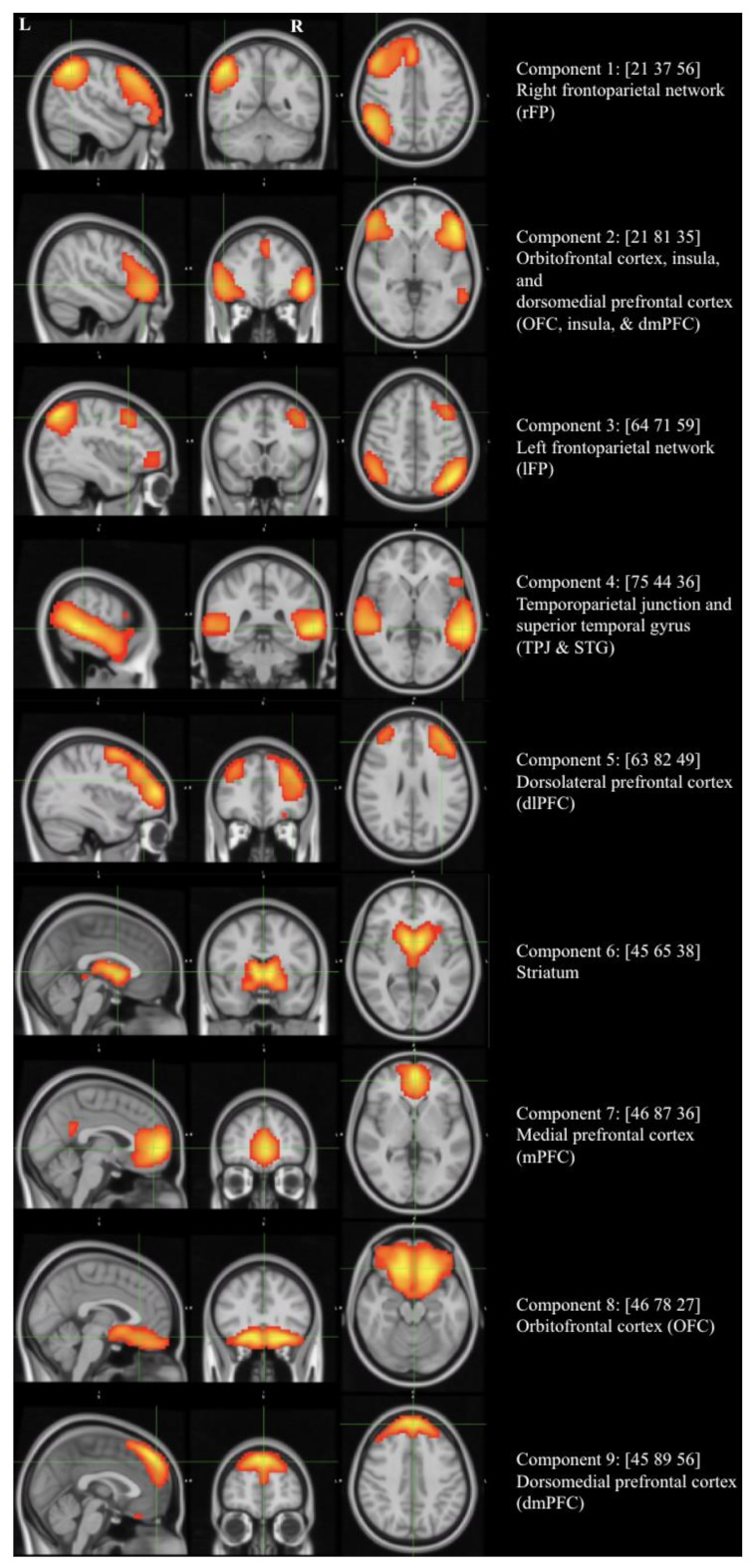
The 9 pre-selected ICA components that included aspects of the OFC, mPFC, striatum, and the FP networks. The coordinates of component slices follow the FSL voxel coordinate system where [x y z] = [0 0 0] refers to the leftmost posterior inferior corner of the brain and [x y z] = [91 109 91] refers to the rightmost anterior superior voxel when the voxel size = 2 × 2 × 2 mm.

From the predefined ICA map [27], we pre-selected nine components that included some portions of the OFC, mPFC, striatum, and the FP networks (see Figure A2). All possible functional connections among these components were tested against measures of persecution via randomization with 5000 permutations for each contrast. The random seed was set to 7 in MATLAB. The p-values were corrected for multiple comparisons so that the family-wise error (FWE) rate was controlled under 0.05.

Using the corrected p-values, we tested the correlations between the strength of resting-state FP–OFC connectivity and the level of persecution, measured by behavioral indices on the MTG and self-reported persecution on the GPTS. We checked the possible effects of handedness (as measured by the Edinburgh Handedness Inventory [29]) and sex on neural correlates associated with persecution.

## 3. Results

### 3.1. Performance and Demographic Characteristics

All measures of persecutory ideation variables were not normally distributed, even with transformation. Thus, we used nonparametric tests (i.e., the Spearman’s correlation test, the Wilcoxon rank sum test, and the Kruskal–Wallis test) whenever possible. The behavioral indices for suspicious mistrust on MTG correlated significantly with the estimated intelligence from WTAR and differed significantly between white and non-white participants (see Table 1). The WTAR raw scores also differed significantly between white and non-white participants (W = 63.5, *p* = 0.001). Unsurprisingly, the MTG percentage of suspicious mistrust and suspicion threshold were highly correlated, but neither were associated with the GPTS score (see Table 1). None of these measures differed significantly among patients with schizophrenia, schizoaffective disorder, mood disorders with psychotic features, or mood disorders without psychotic features (MTG Percentage of Suspicious Mistrust: *p* = 0.099; MTG Suspicion Threshold: *p* = 0.367; GPTS Persecution: *p* = 0.067).

### 3.2. Brain Network Analyses

**Figure 1:**
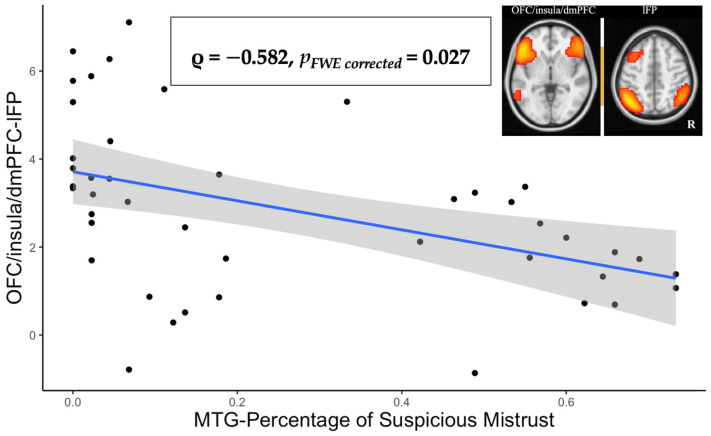
The correlation between MTG Percentage of Suspicious Mistrust and the OFC/insula/dmPFC–lFP connectivity.

Mistrust on the MTG suspiciousness condition, as measured by the likelihood of mistrust, correlated negatively and significantly with the connectivity between the lFP network and the OFC/insula/dmPFC (ρ = −0.582, *p_FWE corrected_* = 0.027; see Figure 1). Curiously, the suspicion threshold showed no significant relationships with any network connections. For example, the correlation between the MTG threshold for suspiciousness and the lFP–OFC/insula/dmPFC connectivity showed a very small effect (ρ = − 0.375, *n.s.*).

Further analyses indicated the relationship between the lFP–OFC/insula/dmPFC time series was generally highly correlated (z = 2.860, *p_FWE corrected_* < 0.001), and, in fact, it was the most correlated of all 36 edges examined. This ruled out the possibility that they were generally uncorrelated and that people who felt persecuted were driving a negative correlation between the regions.

**Figure 2:**
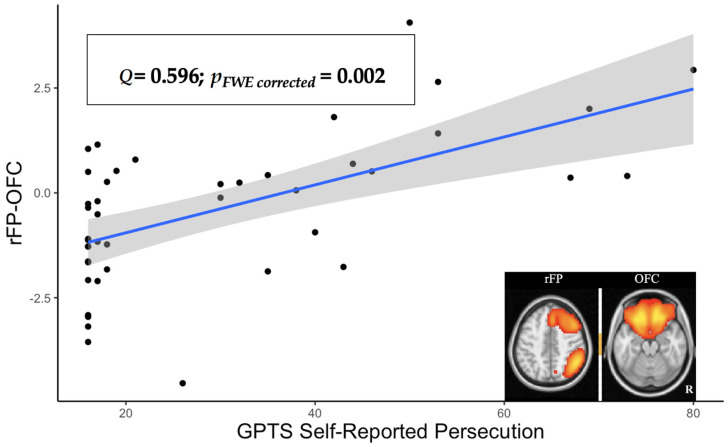
The correlation between the GPTS Self-Reported Persecution and the rFP–OFC connectivity.

To extend these findings beyond behavioral indices, we next considered the self-reported measure of persecutory ideation in relation to the connectivity among the same nine ICA components. Here we found that self-reported persecutory ideations as measured by GPTS correlated significantly with the connection between the right frontoparietal network (rFP) and a different and more concentrated OFC component (ρ = 0.596, two-tailed *p_FWE corrected_* = 0.002; see Figure 2). It should be noted that the rFP–OFC connectivity itself was not significantly different from 0 (z = −0.400, *n.s.*), suggesting that people who felt persecuted showed increases above typically low levels of connectivity.

Handedness did not correlate with either the lFP–OFC/insula/dmPFC (ρ = 0.005; *p* = 0.975) or the rFP–OFC connectivities (ρ = −0.238; *p* = 0.119). Similarly, the strength of the connection did not differ significantly across sex (lFP–OFC/insula/dmPFC: W = 229; *p* = 0.914; rFP–OFC: W = 148; *p* = 0.065).

## 4. Discussion

Wisner, et al. [17] found that more suspicious decisions in the MTG related to reduced connectivity between the lFP networks and the OFC region in a small sample of patients with schizophrenia. One possibility is that this network disconnection represented a functional-structural prior related to patients’ inability to trust an anonymous partner when that partner had a monetary disincentive to betray them. Therefore, to extend these findings, we examined 44 patients receiving services for psychosis-related conditions using a within-group design in a new sample. Controlling for relevant demographic variables, we replicated the finding that lFP to OFC dysconnectivity predicted a higher rate of overall mistrusting decisions (although not necessarily the threshold for those decisions). Furthermore, in an exploratory analysis, the GPTS self-report index of persecutory ideation correlated with increased connectivity between the rFP to OFC networks.

We extended the previous finding of reduced connectivity between the lFP networks and the OFC in patients with elevated persecutory behaviors to the resting state compared to patients who did not behave in a persecuted manner. The lFP network is known to be an integral part of the superordinate cognitive control system [30], alternatively called executive functioning. On the other hand, the OFC is known to be involved in outcome prediction, reward evaluation, and related decision making [31,32,33], and it receives input from the affective relay center of the brain [34]. The significant network found in our study extended from the OFC into the dmPFC, a region associated with social processes [35]. Dodell-Feder et al. [36] found that psychotic patients showed reduced recruitment of the dmPFC during mental state interpretation, while Vargas et al. [37] found that dmPFC efficiency in the mentalizing network was compromised in the clinically at-risk sample for psychosis. Persecutory ideation may be the result of the breakdown of the cognitive control system on social processing and outcome prediction. As OFC continues to receive affective content from other affect-processing regions such as the amygdala, these inputs may require guidance and contextualization from the lFP network. Without this, predictions are likely to be based on affective states rather than broader contextual factors, consistent with the dual process theory [38]. Noticeably, people with high persecutory ideation often reported a high level of negative emotionality as well [39]. In the absence of strong cognitive control, these emotions drive the persecutory interpretations of neutral stimuli [40].

Additionally, we observed a hemispheric difference, such that greater rFP–OFC connectivity predicted elevated self-reported persecutory ideation. While we must interpret this finding cautiously until replicated in a larger, independent sample, this may suggest potential differences between the lFP and rFP’s roles in persecutory social interactions. Considering the low correlation between the behavioral and self-reported measures of persecutory ideation, the two may reflect different aspects of persecutory ideation, differentiating between traits that have less or more demands on patients’ insight.

Lastly, behavioral patterns on the MTG provided a useful measure for persecutory ideation as it reflects on suspicious mistrust specifically as distinct from rational mistrust [17]. The correlations between persecutory ideation and some demographic factors, however, suggest social complexities. The percentage of suspicious mistrust was found to correlate with racial minority status and the WTAR raw score. This translated to more suspicious mistrust behaviors under an economic social decision-making paradigm among racial minority participants, which could be influenced by the social reality of fewer resources and higher discrimination [41]. The association between the WTAR and the racial minority status also suggested that the WTAR raw scores were likely poor estimates for intelligence in a racially diverse patient sample—which was not surprising as performance on such an achievement test would be heavily influenced by one’s amount and quality of education [42].

The primary limitation of the current study was the sample size. While the current sample provides adequate power for many purposes, it can hardly compare to large-scale, cross-institutional neuroimaging projects or large open-data sources. Over the last decade, there has been increased attention to reproducibility in relation to sample size in neuroimaging research. Because of the expected small effect size, neuroimaging research typically requires a large sample size to achieve sufficient power [43]. Marek et al. [44] found a positive relationship between reproducibility and sample size in the existing neuroimaging literature, thus arguing for big sample sizes for stable reproducibility. This study, however, is intended to be a direct response to the concerns about reproducibility, and particularly reproducibility of the previous connectivity findings. We focused our power on a pre-specified group of networks, the constituent voxels of which were selected because they showed ensemble activation, thereby increasing signal-to-noise. Furthermore, this extension of Wisner et al. [17] with a new sample built on somewhat different ICA components. Whereas Wisner’s components were derived from the same sample in which they were tested, the current networks were derived from an independent community sample [27]. Therefore, whereas the associated brain network in Wisner et al. [17] spanned from the OFC to the vmPFC, ours also included parts of the dmPFC and the insula. In addition, the two MTG indices tested were highly correlated with each other. However, they did not correlate with our connectivity metrics consistently. This might be due to the less than ideal distributions of the indices. Further analysis will be needed to optimize the model and to set up indices in a way that maximizes their validity and statistical properties.

Lastly, we had more males than females in our sample, though the ratio (M–F = 28:17) was not unusual for psychosis in clinical settings. We did not have any prior hypothesis for sex differences in neural correlates for persecutory ideation, and we did not find any sex effect on our persecution measures or the functional connectivities associated with those measures. Nonetheless, different patterns of trust have been observed across sex in the general population [45], which may be relevant here.

## 5. Conclusions

The current study provides the first evidence that the functional dysconnectivity between the primary cognitive control region of the brain and a key affective evaluation network relates to suspicious decisions among people with psychosis even when the system is at rest. That is, individual differences in lFP–OFC/insula/dmPFC connectivity seem to act as a functional-structural prior that predisposes some individuals to interpret or react to non-threatening contexts in a suspicious manner, perhaps inferring spiteful intention. While the Minnesota Trust Game is only one approach to measuring such persecutory predispositions, it has a number of advantages, including the use of social decisions with actual monetary consequences. This may be useful for understanding persecutory thought patterns in a guarded population. Furthermore, the use of a within-group design allowed us to focus more closely on a single symptom in our patient participants. We believe this approach can be applied more broadly to take advantage of within-group differences and disentangle the complex heterogeneity found in psychosis.

## Figures and Tables

**Table 1:** Demographics and covariations in the sample of analysis.

	Mean (SD)	Relationship with MTG ^1^ Percentage of Suspicious Mistrust	Relationship with MTG Suspicion Threshold	Relationship with GPTS ^2^ Persecution
N	44	/	/	/
Age	29.7 (7.9)	ρ = −0.037, *p* = 0.814	ρ = 0.029, *p* = 0.850	ρ = 0.169, *p* = 0.273
Sex (% Male)	63.6%	W = 187, *p* = 0.372	W = 187.5, *p* = 0.361	W = 160, *p* = 0.114
% Racial Minority	25%	**W = 288.5, *p* = 0.004**	**W = 289.5, *p* = 0.002**	W = 137, *p* = 0.223
Estimated Intelligence (WTAR ^3^ Raw Score)	38.6 (8.7)	**ρ = −0.392, *p* = 0.009**	**ρ = −0.403, *p* = 0.007**	ρ = −0.111, *p* = 0.475
Education (yrs)	15.5 (2.3)	ρ = −0.249, *p* = 0.108	ρ = −0.229, *p* = 0.139	ρ = −0.023, *p* = 0.886
Parental Education (average yrs)	16.0 (3.5)	ρ = −0.134, *p* = 0.393	ρ = −0.154, *p* = 0.324	ρ = −0.092, *p* = 0.555
Handedness (1 = left; 5 = right)	4.2 (0.9)	ρ = 0.105, *p* = 0.498	ρ = 0.214, *p* = 0.164	ρ = −0.151, *p* = 0.329
MTG Percentage of Suspicious Mistrust	25.8% (26.6%)	-	-	-
MTG Suspicion Threshold	−5.7 (11.3)	**ρ = 0.882, *p* < 0.001**	-	-
GPTS Persecution	29.6 (18.1)	ρ = 0.161, *p* = 0.296	ρ = 0.087, *p* = 0.577	-

^1^ MTG = Minnesota Trust Game [24] ^2^ GPTS = Green et al. Paranoid Thoughts Scales [25]; ^3^ WTAR = Wechsler Test of Adult Reading [21]. **Bold** text indicates significant correlations.

## Data Availability

The data presented in this study are available on request from the corresponding author. The data are not publicly available because explicit permission was not given by participating patients.
